# Regrowth of Mycobacterium tuberculosis Populations Exposed to Antibiotic Combinations Is Due to the Presence of Isoniazid and Not Bacterial Growth Rate

**DOI:** 10.1128/AAC.00570-19

**Published:** 2019-11-21

**Authors:** Charlotte L. Hendon-Dunn, Henry Pertinez, Alice A. N. Marriott, Kim A. Hatch, Jon C. Allnutt, Geraint Davies, Joanna Bacon

**Affiliations:** a TB Research Group, National Infection Service, Public Health England, Salisbury, Wiltshire, United Kingdom; b Department of Molecular and Clinical Pharmacology, University of Liverpool, Liverpool, United Kingdom; c Institute of Infection and Global Health, University of Liverpool, Liverpool, United Kingdom

**Keywords:** *Mycobacterium tuberculosis*, antagonism, antibiotic combinations, bactericidal activity, chemostat culture, growth rate, isoniazid, mathematical modeling, pyrazinamide, relapse

## Abstract

Modulation of the growth rate in Mycobacterium tuberculosis is key to its survival in the host, particularly with regard to its adaptation during chronic infection, when the growth rate is very slow. The resulting physiological changes influence the way in which this pathogen interacts with the host and responds to antibiotics. Therefore, it is important that we understand how the growth rate impacts antibiotic efficacy, particularly with respect to recovery/relapse. This is the first study that has asked how growth rates influence the mycobacterial responses to combinations of the frontline antimycobacterials, isoniazid (INH), rifampin (RIF), and pyrazinamide (PZA), using continuous cultures. The time course profiles of log-transformed total viable counts for cultures, controlled at either a fast growth rate (mean generation time [MGT], 23.1 h) or a slow growth rate (MGT, 69.3 h), were analyzed by the fitting of a mathematical model by nonlinear regression that accounted for the dilution rate in the chemostat and profiled the kill rates and recovery in culture. Using this approach, we show that populations growing more slowly were generally less susceptible to all treatments. We observed a faster kill rate associated with INH than with RIF or PZA and the appearance of regrowth. In line with this observation, regrowth was not observed with RIF exposure, which provided a slower bactericidal response. The sequential additions of RIF and PZA did not eliminate regrowth. We consider here that faster, early bactericidal activity is not what is required for the successful sterilization of M. tuberculosis, but instead, slower elimination of the bacilli followed by reduced recovery of the bacterial population is required.

## Abstract (precis)

Modulation of the growth rate in Mycobacterium tuberculosis is key to its survival in the host, particularly with regard to its adaptation during chronic infection, when the growth rate is very slow. The resulting physiological changes influence the way in which this pathogen interacts with the host and responds to antibiotics. Therefore, it is important that we understand how the growth rate impacts antibiotic efficacy, particularly with respect to recovery/relapse.

## INTRODUCTION

Improvement in tuberculosis (TB) treatment with the aim of shortening the period of antibiotic therapy without increasing relapse rates or encouraging the development of antibiotic-resistant strains is urgently needed (1). Although new combinations of antibiotics with novel modes of action are being evaluated, the optimal dosing and treatment duration can be investigated further for existing antibiotics. Understanding how the growth rate impacts the activity of frontline antibiotics so that they can be delivered using an alternative approach or in a different timely fashion can contribute to the development of regimens that contain a combination of both conventional and new antibiotics (2–5).

One of the requirements of a new antibiotic combination is its ability to target heterogeneous populations of bacteria, particularly those that develop antibiotic tolerance during the course of treatment and that are thought to contribute to relapse posttreatment (6). Fluctuations in the growth rate in Mycobacterium tuberculosis enable the organism to adapt to different environmental niches in the host, particularly with regard to its adaptation during chronic infection, when the growth rate becomes very slow. The resulting genotypic and phenotypic changes influence the way in which this pathogen interacts with the host and responds to antibiotic treatment. Therefore, it is important that we understand how growth rate impacts antibiotic efficacy. The current paradigm concerning the effect of growth rate or growth phase on the response of M. tuberculosis to antibiotic therapy relies heavily upon the notion that fast-growing bacteria are more susceptible to the action of antibiotics than slow growers (7–10). The slow-growing proportion of the M. tuberculosis population is thought to be refractory to the bactericidal action of antituberculosis antibiotics due to phenotypic tolerance and persistence through treatment (10). Informative *in vitro* evaluations using batch models have determined the activity of antibiotics against relevant phenotypes, including nonreplicating persistent bacteria (11–14). However, it is challenging to dissect out the direct cause and effect of a single stimulus in batch cultures. The only models that can be used to determine the effects of the growth rate on drug responses are controlled and defined continuous cultures in chemostats (14–17). These growth systems enable us to control the growth rate with minimal changes to the physiochemical environment, thereby allowing the effects of different growth rates to be compared. In this study, we investigated the contribution of growth rate to early bactericidal kill and the regrowth/recovery of the bacterial population when exposed to isoniazid (INH), pyrazinamide (PZA), and rifampin (RIF), delivered singly or in combination. To enable us to perform these analyses, we have derived a mathematically discriminative approach for the analyses of drug responses in chemostat culture which accounts for dilution effects and provides predictive and quantitative insights from the bacterial responses.

## RESULTS

The antibiotics INH, RIF, and PZA were added during culture steady state at the MICs to replicate cultures for each single antibiotic or antibiotic combination at each growth rate, except for the triple combination, for which a single culture was performed at each growth rate. A static concentration of antibiotic was maintained in culture throughout each time course. Viable counts were performed throughout the culture time courses and for a minimum of 14 mean generation times (MGT), which is equivalent to 970.2 h and 322.2 h for slow growth and fast growth, respectively. The time course profiles of the log-transformed viable counts for each culture were analyzed with the fitting of a mathematical model by nonlinear regression, where parameters representing gradients on the logarithmic scale were determined for each culture to describe the logarithmic transformation of the viable count profile over time. These overall gradients comprise an estimated *k*_net_ rate constant parameter that accounts for the net bacterial death/regrowth and a fixed *k*_chemo_ rate constant imposed by the chemostat culture according to fast growth or slow growth conditions (equations 1 and 2). A minimum of at least one gradient is needed to describe a log viable count profile: the initial α gradient, typically indicating a bactericidal or a bacteriostatic response, where α is equal to (*k*_net_α_ − *k*_chemo_). If more than the α gradient is needed to adequately describe the log viable count profile (i.e., if more than one exponential phase is present), then additional gradient parameters are estimated accordingly, where β is equal to (*k*_net_β_ − *k*_chemo_). Additional gradients indicate responses other than the initial net kill, e.g., regrowth/recovery at a faster net growth rate, net kill at a rate different from that initially observed, or a reestablished steady-state growth. The *k*_net_α,_ and *k*_net_β_ elimination rate constants were compared between pairs of culture conditions to determine the impact of the growth rate in each treatment case, while accounting for the chemostat dilution rate, *k*_chemo_, for each growth rate (Table 1). *P* values from pairwise *Z*-tests comparing relevant pairs of experiments were calculated (Table 2) to determine which growth rate-specific responses were significantly different.

**TABLE 1:** Values for *k*_net_α_, *k*_net_β_, and *k*_net_γ_ (if present) with the percent error for slow-growing or fast-growing continuous cultures of Mycobacterium tuberculosis exposed to INH, RIF, and PZA, singly and in combination

Treatment	Slow-growing continuous cultures (*k*_chemo_ = −0.01)						Fast-growing continuous cultures (*k*_chemo_ = −0.03)					
	*k*_net_α_ (h^−1^)		*k*_net_β_ (h^−1^)		*k*_net_γ_ (h^−1^)		*k*_net_α_ (h^−1^)		*k*_net_β_ (h^−1^)		*k*_net_γ_ (h^−1^)	
	Est.^*a*^	% RSE	Est.	% RSE	Est.	% RSE	Est.	% RSE	Est.	% RSE	Est.	% RSE
RIF	0.0001^*b*^	288^*b*^					−0.020	26				
INH	−0.041	52	0.013	10			−0.118	47	0.033	7		
RIF and INH	−0.030	19	0.013	22			−0.102	18	0.029	37		
RIF, INH, and PZA	−0.036	55	0.007	44			−0.010	38				
INH at 16× MIC	−0.110	35	−0.003	13	0.014	4	−0.104	19	−0.009	69	0.037	6
Control	0.013	9.12					0.031	4.3				

a Est., estimate.

b The *k*_net_α_ estimate is very close to 0; i.e., the overall observed α elimination rate of the viable count is approximately equal to the *k*_chemo_ washout rate, leading to an inflated relative standard error of this estimate.

**TABLE 2:** *P* values for pairwise comparisons of *k*_net_α_ in fast-growing and slow-growing continuous cultures of M. tuberculosis exposed to MICs of INH, RIF, and PZA singly and in combination^*a*^

Pairwise comparison	*P* value
*k*_net_α_ comparisons	
RIF, slow vs fast	<0.01
INH, slow vs fast	0.20
RIF-INH, slow vs fast	0.02
RIF-INH-PZA, slow vs fast	1.34
INH at 16× MIC, slow vs fast	1.00
RIF vs INH, slow	0.84
RIF vs RIF-INH, slow	<0.01
INH vs RIF-INH, slow	1.34
RIF vs INH, fast	0.08
RIF vs RIF-INH, fast	<0.01
INH vs RIF-INH, fast	0.80
RIF-INH vs RIF-INH-PZA, slow	1.28
RIF-INH vs RIF-INH-PZA, fast	<0.01
INH vs INH at 16× MIC, slow	0.12
INH vs INH at 16× MIC, fast	0.81
RIF vs RIF-INH-PZA, slow	0.06
INH vs RIF-INH-PZA, slow	1.11
RIF vs RIF-INH-PZA, fast	0.49
INH vs RIF-INH-PZA, fast	0.07
*k*_net_β_ and *k*_net_γ_ comparison	
RIF-INH vs RIF-INH-PZA, slow	1.18

a Fast-growing continuous cultures (fast) had an MGT of 23.1 h, and slow-growing continuous cultures (slow) had an MGT of 69.3 h. *k*_net_α_ comparisons were also made for 16× MIC INH, and *k*_net_β_ and *k*_net_γ_ comparisons were made for responses that were triphasic.

### The rate of kill was higher in fast-growing M. tuberculosis.

**FIG 1:**
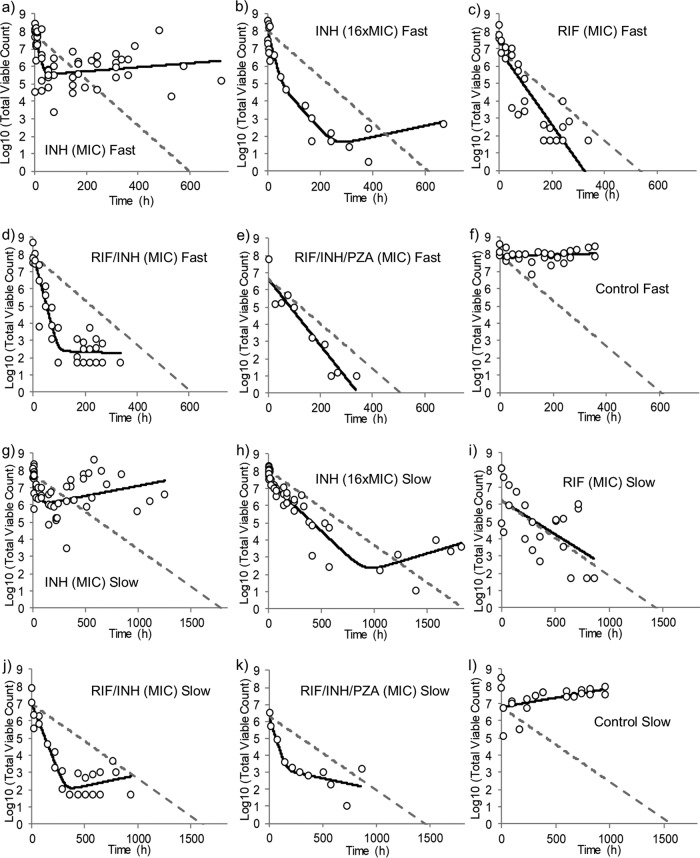
Viability of M. tuberculosis growing under a fast growth rate (MGT, 23.1 h) (a to f) or a slow growth rate (MGT, 69.3 h) (g to l) in continuous culture and exposed to either INH at 0.5 mg ml^−1^ (a, g), INH at 8 mg liter^−1^ (b, h), RIF at 0.032 mg liter^−1^ (c, i), INH at 0.5 mg liter^−1^ and RIF at 0.032 mg liter^−1^ (d, j), INH at 0.5 mg liter^−1^, RIF at 0.032 mg liter^−1^, and PZA at 250 mg liter^−1^ (e, k), or no antibiotic as a control (f, l). Total viable counts (number of log_10_ CFU per milliliter; circles) were determined by plating; the mathematical model, governed by the estimated *k*_net_α/β_ and intercept parameters (solid black line), was fitted to the data; and the underlying imposed chemostat washout rate (gray dashed line) was used as a comparison.

Early bactericidal activity was observed in all cultures (as shown by negative *k*_net_α_ kill rate constants; Table 1), except for bacteriostatic responses to single RIF exposures in slow growers (where *k*_net_α_ was close to 0; Fig. 1i). Slow-growing cultures exposed to INH at the MIC, either singly or in combination (Fig. 1g, j, and k), all showed initial killing rates similar to each other (with *k*_net_α_ values ranging from −0.03 to −0.04). Apart from the results obtained with the RIF-INH-PZA triple combination (*k*_net_α_ = −0.036 and −0.01 for slow growth and fast growth, respectively), the fast growers showed an initial net bacterial killing rate of a greater magnitude than the slow growers for any given antibiotic therapy. Fast growers or slow growers exposed to RIF alone (Fig. 1c and i) and fast growers exposed to the triple combination (Fig. 1e) demonstrated monophasic elimination over time, with the kill rates being lower than those for all INH- and INH-RIF-exposed cultures (Fig. 1a, d, g, and j; Table 1).

The further addition of PZA to the RIF-INH combination in a slow-growing culture (Fig. 1k) resulted in a kill rate similar to that of INH used singly (Fig. 1g) and the RIF-INH dual combination (Fig. 1j) (*P = *1.11 and 1.28, respectively), indicating that there were no additional killing benefits by the addition of PZA. However, when making a similar comparison in a fast-growing culture, the kill rate for RIF-INH-PZA (Fig. 1e) was lower than that for the double combination (Fig. 1d) and INH single treatment (Fig. 1a) (*k*_net_α_ = −0.01 for the triple combination versus *k*_net__α = ∼−0.1 [*P = *0.07 for INH, *P < *0.01 for RIF-INH]), indicating that the further addition of PZA to the fast-growing population reduced the beneficial effects of INH killing. This was indicative of a potential antagonistic effect between INH-RIF and PZA.

### Increasing the INH concentration does not eliminate regrowth.

Increasing the exposure of INH from the MIC to a 16-fold higher MIC (8 mg liter^−1^, a concentration more reflective of patient serum levels in the clinic) gave rise to a faster initial killing rate in slow-growing cultures (*k*_net_α_, −0.041 versus −0.110 [*P = *0.12] for the MIC versus 16× MIC, respectively) (Fig. 1g and h). However, the concentration increase made little difference to the initial killing rate in fast-growing cultures (*k*_net_α_, −0.118 versus −0.104 [*P = *0.81] for the MIC versus 16× MIC, respectively) (Fig. 1a and b), perhaps suggesting that the kill rate for INH at the MIC is maximal for fast-growing bacteria and indicating that fast-growing bacteria are more susceptible to INH. The responses to 16× MIC INH exposure were also the only trends that followed a triphasic profile, with two distinguishable initial bactericidal phases, indicating that the higher INH concentration provoked a response in the bacterial population different from that provoked by INH at the MIC. As discussed above, in all four of these situations (fast or slow culture with the MIC or 16× MIC INH), a regrowth/recovery phase was observed, with the regrowth rate being approximately equivalent in size to the dilution rate for each culture, leading to relatively flat second and final phases to the viable count profiles, implying a reestablishment of steady state. However, at the higher INH concentration, for both growth rates, the apparent time of onset of the net regrowth/recovery phase was later and occurred from a starting point of a lower viable count.

### Regrowth in response to INH is not eliminated by PZA and/or RIF.

The addition of RIF to INH did not alter the kill rate or the regrowth rate compared to the rates achieved with INH alone for the fast growers or the slow growers. Regrowth in the presence of the RIF-INH combination was apparent after 100 h postexposure in fast-growing cultures (Fig. 1d) and after approximately 350 h in slow growers (Fig. 1j), with *k*_net_β_ values being 0.029 and 0.013, respectively. The addition of PZA in the triple combination of PZA-RIF-INH eliminated regrowth in the fast-growth-rate culture (Fig. 1e). In slow-growing cultures, there was a reduction in the regrowth rate (from *k*_net_β_ = 0.013 to *k*_net_β_ = 0.007). However, regrowth was not eliminated entirely. What was surprising was a lack of a reduction in the kill rate in the slow-growth culture (Fig. 1k) with the addition of PZA. These findings show that PZA appears to have more of an effect in reducing the regrowth of fast-growing cultures than that of slow growers. The only situation in which a combination containing INH did not result in regrowth was for the fast-growing culture treated with RIF-INH-PZA. It is possible that the lower kill rate in the fast growers exposed to the triple combination contributed to a lack of regrowth, as this was also observed for RIF exposure, where the killing rate was also more gradual.

## DISCUSSION

### The rate of kill was faster in fast-growing M. tuberculosis.

Apart from the triple combination, for any given antibiotic treatment, there was a more rapid initial bacterial killing in fast growers than in slow-growing cultures. It is widely accepted that bacteria in stationary phase/nonreplicating phases are less susceptible *in vitro* to the frontline antibiotics RIF and INH (12, 13, 18, 19). However, the effect of growth rate on the effectiveness of drug combinations has never been looked at in M. tuberculosis and is particularly important for organisms that can survive in the host for long periods of time under slow-growing or nongrowing conditions, which are characteristic of TB disease progression. Some of these studies have included the use of mathematical models, which described three bacterial states, representing fast-multiplying, slow-multiplying, and nonmultiplying bacteria, in order to identify the responses to antibiotics (12). These mathematical models have predicted exposure-response relationships by inferring bacterial cell states within batch cultures, where interpretation of drug responses was confounded by many factors other than growth rate. Continuous culture provides an invaluable method for determining growth rate effects while controlling other parameters in the system (14, 15, 17). A limitation of these systems is that the interpretation of bacterial responses is complicated by the continuous flow of medium into the chemostat and the loss of cells in the effluent, particularly as this dilution rate is different for the two growth rates. We have accounted for this limitation in the mathematical model described here. Antibiotic activity in other bacteria is also impacted by the growth rate, as shown in previous experiments using the chemostat, but very few of these studies have used mathematical models to quantify and determine the bacterial responses (16, 17, 20, 21). Levin and Udekwu (22) developed a quantitative model framework, using parameter values from the literature (or best *a priori* estimates), for generating simulations, hypotheses, and interpretations of bacterial responses in batch or continuous cultures. This model accounts for culture dilution rates and the drug dilution rate (drug clearance in pharmacokinetic terms) and described various considerations that could be incorporated into future experimental designs, such as the effect of secondary resources from dead cells and metabolites that have been released, wall subpopulations (biofilms), and the impact of reduced cell density on the MIC. Other plausible mathematical models could be used to describe these phenotypic responses and bacterial population dynamics. However, given that we observed viable count data rather than any quantification of specific subpopulations and antibiotic exposures were performed at one or two drug concentrations, more complex mathematical models were not applied at this stage.

### Increasing the concentration of INH does not remove regrowth.

Exposure to INH in the chemostat led to rapid bactericidal responses followed by regrowth/recovery, and this was irrespective of the growth rate. Recovery in the chemostat was attributable to a population of bacteria that either increased their growth rate or maintained a growth rate that was dictated by the flow rate of the growth medium (14). These experiments indicate that biexponential killing and persistence through INH exposure in clinical or *in vivo* studies are not explained by a reduced growth rate. We previously showed under both these growth rates that recovery was coincident with a substantial increase in mutant frequency and that the bactericidal activity of INH was being arrested, in part, by the emergence of *katG* resistance mutations and not a reduction in the log-phase populations, irrespective of the growth rate (14, 23). This finding was also reflected in another study by Gumbo et al., in 2007, using a hollow-fiber pharmacodynamic model (24). However, we also showed that the response to INH alone is not entirely explained by an increase in mutant frequency but is also explained by substantial genotypic changes that are growth rate specific (14). The biphasic kill observed in M. tuberculosis-infected guinea pigs treated with INH was found to be associated with the emergence of antibiotic-tolerant persisting populations that were not resistant mutants (25). Similarly, relapse has previously been observed in mice after the cessation of treatment. Of all treatment groups that included INH, at least 30% of the mice relapsed (as defined by the isolation of M. tuberculosis from the spleen), whereas in treatment groups without INH, tubercle bacilli could be isolated from only 8% of mice (26). The lack of reduced recovery at higher concentrations has also been observed previously over a range of INH concentrations both *in vitro* and *in vivo* (13, 27).

### Addition of RIF and PZA does not eliminate the regrowth associated with INH.

Despite the response to RIF being slower and monophasic, it was unable to remove the recovery elicited by INH when used in a combined exposure at either growth rate. Similarly, a lower rate of kill by RIF has previously been shown *in vitro* and in mice, with no relapse being observed (27). These findings were also reflected in a study by Hu et al., in 2016, where RIF-containing regimens reduced persistence in mice (19). These findings, combined with the fact that RIF has recently been shown to be effective at higher doses, indicates that the removal of INH in treatment and the use of an increased dose of RIF are worthy of investigation as attractive options for new drug combinations (2, 28–30). It would be interesting to see if increasing the concentration of RIF in our chemostat system leads to changes in the bacteriostatic versus bactericidal response and whether this is growth rate specific or not. A murine study by Andries et al. in 2010 ranked the bactericidal and sterilizing potencies of several regimens (and individual antibiotics), and the comparison of the two ranks highlighted that bactericidal activity is not predictive of sterilization (31). Their study also confirmed that RIF possessed more sterilizing activity than expected from the bactericidal efficacy, further emphasizing the need to move away from early bactericidal activity as a priority measure of drug candidate potential. A new drug combination needs to have good sterilizing potential for genuine clinical cure/efficacy, but prediction of this is challenging. Improved bacterial markers of drug-tolerant persistent subpopulations that can be measured early in the development of a drug combination are required, and progress has been made in this area (32). Our results also suggest that the addition of PZA in the triple combination reduced the beneficial effects of INH killing of fast-growing bacteria, indicating that there could be antagonism between PZA and INH in chemostat culture. PZA could be bacteriostatic against the fast growers, resulting in a halt in replication, which in turn reduced the fast killing effects of INH (33). It has been shown that growth inhibition by bacteriostatic antibiotics is associated with suppressed cellular respiration, whereas cell death from exposure to bactericidal antibiotics causes accelerated respiration (34). A reduced cellular respiration induced by PZA exposure could be an explanation for the antagonism observed between INH and PZA. It may be that the slower INH killing of fast growers leads to a lack of regrowth. INH has also been shown to have an antagonistic effect on the efficacy of PZA in mice (19, 27, 35, 36), providing further support for the removal of INH either entirely or after an appropriate duration of treatment, and it would therefore be interesting to see, using our continuous culture models, whether the removal of INH after 1 to 2 days (with continued exposure to PZA and RIF) reduces the presence of regrowth.

### Concluding remarks.

Regrowth is associated with the presence of INH and not the bacterial growth rate. The association between INH and recovery/relapse, combined with the knowledge that M. tuberculosis overcomes the effects of INH after an initial killing phase by the development of resistance and/or drug tolerance in any *in vitro* or *in vivo* model, supports the suggested replacement of INH with drugs that kill more slowly and that do not lead to a relapse. The chemostat model and the method of data analysis described here can contribute to the direct comparison of drug combinations and provide information about the relationship between bactericidal activity and recovery.

## MATERIALS AND METHODS

### Reagents.

Primary stock solutions of RIF, INH, and PZA were prepared at 10 g liter^−1^ in 100% dimethyl sulfoxide. These were frozen in aliquots (100 μl) at −20°C. A working stock of RIF was prepared at 1 g liter^−1^ (diluted from the 10-g liter^−1^ stock using water) and was also frozen at −20°C. When required, the stocks were diluted to the desired concentration in water and filter sterilized (0.2-μm pore size).

### Strains and their growth.

M. tuberculosis (strain H37Rv) was used in all experiments. Bacilli were enumerated on 7H10 agar plus oleic acid-albumin-dextrose-catalase supplement.

### Continuous culture of M. tuberculosis.

M. tuberculosis (strain H37Rv) was grown in chemostats under controlled conditions as described previously (14). We cultured M. tuberculosis using CAMR *Mycobacterium* medium MOD2, which contains glycerol as the limiting nutrient (37). Continuous cultures were performed at two different growth rates to steady state under defined and controlled conditions at pH 6.9, at a temperature of 37°C, and at a dissolved oxygen tension of 10% (14). The cultures achieved an MGT of 23.1 h (fast growth) or an MGT of 69.3 h (slow growth), where the fractional washout/replacement rate of the medium in the continuous culture was 0.03 h^−1^ and 0.01 h^−1^, respectively. Antibiotics were added during steady state at the MICs (0.5 mg liter^−1^ of INH, 0.032 mg liter^−1^ of RIF, and 250 mg liter^−1^ of PZA) to two replicate cultures for each single antibiotic or antibiotic combination at each growth rate except for the triple combination, where a single culture was performed at each growth rate. A static concentration of antibiotic was maintained in culture throughout each time course. In the case of INH, exposures to 16× MIC (8 mg liter^−1^) were also assessed. Viable counts were performed throughout the culture time courses and for a minimum of 14 MGT, which is equivalent to 970.2 h and 322.2 h for slow growth and fast growth, respectively (14). Further triplicate fast-growth or slow-growth cultures were established without antibiotic exposure to provide baseline information about the differences in viability between the two growth rates.

### Viability measurements.

The viability of the cultures was measured at each MGT using the viable count method of Miles et al. (38) with the following modification: the plate was divided into quadrants for the dilutions. In each quadrant, three 20-μl aliquots of the appropriate dilution were spotted, and then the plates were left to dry at room temperature. Colonies were counted after 3 weeks of incubation at 37°C.

### Mathematical modeling.

The growth rate, death rate, and chemostat washout rate (by continuous culture) of a given population of M. tuberculosis in the continuous culture incubation were described mathematically as first-order processes, i.e., processes where the rates of bacterial growth, death, or chemostat elimination in the continuous culture at a given instant of time were all proportional to the size of that population of M. tuberculosis at that given instant of time and governed by the first-order rate constants *k_g_*, *k_d_*, and *k*_chemo_ for the growth, death, and chemostat washout, respectively. When they were combined, these component rates give an expression for the overall rate of change of the size of the M. tuberculosis population (MTb) in log_10_ CFU ml^−1^ (equation 1):

(1) $$d\left(\text{MTb}\right)/\mathrm{dt}=\left(k_{g}\times \text{MTb}\right)–\left(k_{d}\times \text{MTb}\right)–\left(k_{\text{chemo}}\times \text{MTb}\right)$$

where *t* is time.

Equation 1 can be simplified as outlined in equation 2:

(2) $$d\left(\text{MTb}\right)/\mathrm{dt}=\left(k_{g}–k_{d}–k_{\text{chemo}}\right)\times \text{MTb}$$

$$=\left(k_{\text{net\_α}}–k_{\text{chemo}}\right)\times \text{MTb}$$

=α×MTb

Equation 2 summarizes that the rate of change of the M. tuberculosis population was governed by an overall net first-order rate constant, α, itself comprised of a net bacterial growth/death rate constant (*k*_net_α_) and the chemostat continuous fractional washout rate constant (*k*_chemo_; fixed to 0.01 h^−1^ and 0.03 h^−1^ for slow-growing or fast-growing cultures, respectively. Equation 2 (an ordinary differential equation) was integrated into an exponential, closed-form solution describing the M. tuberculosis population as a direct function of time and the initial size of the population (equation 3):

(3) MTb(time)=A×exp(α×time)

where *A* is the initial size of the M. tuberculosis population.

In this form, α is the gradient of a plot of the natural log transform of the count of the M. tuberculosis population versus time or the apparent gradient of a plot of the M. tuberculosis population versus time on a semilog scale and has units of time^−1^. *A* is the *y* axis intercept of the same transformed profile/plot. This mathematical approach allows for a more precise partitioning of the overall behavior of the bacterial population over time (seen at the level of the raw data via the α rate constant) into the processes of bacterial growth/death (governed by *k*_net_α_) and the fixed, known, and experimentally controlled process of the chemostat continuous culture washout (governed by *k*_chemo_). If it is apparent from a plot of the logarithmic transformation of the observed viable count data that more than one exponential phase (i.e., more than one gradient on the log scale) is required to describe the observations, then equation 3 (and its underlying assumptions) can be doubled up into a biexponential equation (equation 4).

(4) MTb(time)=A×exp(α×time)+B×exp(β×time)

**FIG 2:**
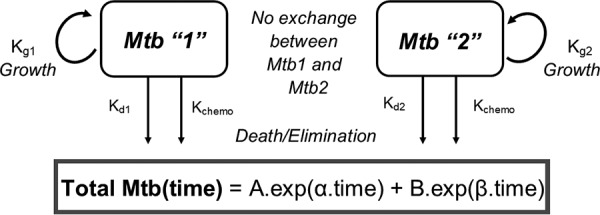
A biexponential, two-state mathematical model applied to viable count data obtained from continuous cultures of Mycobacterium tuberculosis that were treated with static concentrations of INH, RIF, and PZA either singly or in combination. Total Mtb = MTb_1_ + MTb_2_, α = *k_g_*_1_ − *k_d_*_1_ − *k*_chemo_ = *k*_net_α_ − *k*_chemo_, β = *k_g_*_2_ − *k_d_*_2_ − *k*_chemo_, and *k*_chemo_ = *k*_net_β_ − *k*_chemo_. *k_g_*, bacterial replication (growth); *k_d_*, bacterial death; *k*_chemo_, bacterial washout due to dilution rate of chemostat. The sign and magnitude of *k*_net_ typically depend on *A* or α, which governs the kill phase of antibiotic treatment, and *B* or β, which governs the regrowth phase of antibiotic treatment. A two-state model was adequate to describe the data set profiles for most cultures. However, some cultures demonstrated single or triple exponential phases and required either one state or three states for the data to be adequately described.

Here, *B* and β represent the initial value and the overall net first-order rate constant describing the behavior of a 2nd subpopulation of M. tuberculosis contributing to the total observed viable count, respectively. This second subpopulation may have had a slower or faster overall net growth or death rate than the other and/or may have begun from a different initial starting point. This second subpopulation may have contributed to potential regrowth that was not obvious until later in the time course or perhaps to a second slower elimination phase under drug therapy. Further exponential terms (e.g., using the parameters *G* and γ) may be added if the data have extra phases able to support their estimation. A schematic of the processes considered by the overall mathematical model in equation 4 applied in this work is given in Fig. 2. The mathematical models of equation 3 or equation 4 were applied to the time course profiles of viable counts from continuous culture to provide an estimate of the α elimination rate and, if present, the β regrowth rate and/or their corresponding *k*_net_α_ or *k*_net_β_ values, given the known, fixed *k*_chemo_ washout rate constant. These parameter estimates compare net growth or elimination rates between different experimental conditions and are used to simulate a line of best fit to provide a visual check of how effectively the mathematical model describes the data. Observations of the minimum viable count in culture were also compared to the initial value at the start of treatment to estimate an observed maximum reduction in the viable count, to be used in conjunction with the mathematical model parameter values estimated from the data to provide any extra insight into drug activity. Parameter estimation from applying the model to the data used the nonlinear least-squares optimization function lsqnonlin as part of the pracma package in the R statistical software language (version 3.4.2) with an unweighted objective function. The standard errors (SE) of the parameter estimates were calculated using the method outlined by Landaw and DiStefano, with the Jacobian of model parameter sensitivities being estimated using a numerical central difference method (39). SE were expressed as a percentage relative to the parameter estimate in question (percent relative standard error [RSE), with an RSE of less than ∼50% being considered an acceptable degree of precision of an estimate, given the data in question. The replicate experiments were treated as a naive pool for data analyses, rather than using the average of replicate data at each time point (40). The significance of the differences between model parameter estimates under different experimental conditions was examined with pairwise *Z*-tests.

### Interpretation of *k*_net_α_ and *k*_net_β_ values.

If gradients *k*_net_α_, *k*_net_β_, and *k*_net_γ_ were <0, then the net rate of change of the M. tuberculosis population is negative and a plot of the M. tuberculosis population viable count in the continuous culture versus time would show an exponential decrease over time. This is the case for all negative *k*_net_ values, *k*_net_ equal to 0 (i.e., bacteriostasis), or any positive *k*_net_ values that are not large enough in magnitude to cancel out the imposed, fixed *k*_chemo_ (i.e., net growth, but not fast enough to completely overcome the chemostat washout rate). A negative gradient value is therefore indicative of either bacteriostatic or bactericidal activity. If a gradient is equal to 0, then the net rate of change of the M. tuberculosis population is zero and a plot of the population log viable count versus time will be flat. For this situation, *k*_net_ must equal (−1) × *k*_chemo_. A gradient of 0 (or a value that is very close to 0) indicates growth equal to the imposed rate of chemostat washout, as seen in the steady-state control, no-drug cultures. Estimates of *k*_net_ that are very close to 0 can show poor relative standard error precision for the parameter estimate. If the gradient is >0, then the net rate of change of the M. tuberculosis population is positive and a plot of the population viable count versus time shows an exponential increase over time. For this, *k*_net_ must be both positive and larger in absolute value than *k*_chemo_. A positive gradient is therefore an indication of regrowth/recovery or of an M. tuberculosis population in the overall culture.
